# The patient journey and guideline-concordant care in colorectal cancer—is current practice enough?

**DOI:** 10.3389/fmed.2026.1810895

**Published:** 2026-04-09

**Authors:** Carlton C. Barnett, Gavin R. Oliver, Michael Rutenberg Schoenberg, Hans P. Smith, W. Roy Smythe

**Affiliations:** 1 Xilis, Inc., Durham, NC, United States; 2 University of Colorado School of Medicine, Aurora, CO, United States

**Keywords:** colorectal cancer, functional precision medicine, guideline concordant care, metastasis, minimal residual disease, new approach methodologies, resistant subclones, tumor heterogeneity

## Abstract

Colorectal cancer is a major healthcare burden, and modern management of non-metastatic disease largely depends on guideline-concordant care based on histopathologic staging and empiric systemic therapy. While multidisciplinary care pathways and standardized guidelines have improved outcomes at the population level, they fall short in addressing the inter-patient and intra-tumoral heterogeneity that contributes to treatment resistance, recurrence of the disease, and unnecessary toxicity. Using a hypothetical patient journey, this commentary highlights how current practice often fails to align with patient needs despite being guideline-concordant. We discuss the limitations of current treatment paradigms and the shortcomings of modern tools such as genomic profiling, highlighting the continued need for complementary approaches. We hypothesize that functional precision medicine approaches have the potential to complement existing treatment paradigms and improve therapeutic stratification. We provide illustrative examples of their potential utility drawn from our recent clinical correlation study on colorectal cancer, in which we reported an association between assay outcomes and clinical response in a retrospective cohort. Additionally, we demonstrate the ability to identify intra-patient heterogeneity in *ex vivo* drug responses, suggesting phenotypically distinct subpopulations with differential drug sensitivity. Further investigation leading to the integration of these or similar technologies alongside genomic and minimal residual disease assessments could refine therapy selection and improve existing surveillance strategies. Ultimately, we suggest that while guideline-concordant care remains necessary, it is not sufficient for all patients. Continued research efforts utilizing functional precision medicine technologies may enable colorectal cancer management to move toward a more personalized framework that maximizes patient outcomes.

## 1 Introduction

Colorectal cancer represents a considerable health burden worldwide, being the third most common cancer type and the second-leading cause of cancer-related death (1). The standard of care for patients with non-metastatic colorectal cancer is surgical resection, with clinical and histopathologic staging informing the recommendation for adjuvant systemic therapy (2, 3).

Currently, there are no validated markers that can reliably guide chemotherapy selection in patients for whom histopathologic staging indicates that systemic therapy may be beneficial (4, 5). Further, the toxicity of systemic therapy is of great concern to clinicians and patients alike (6).

Despite recent enthusiasm for a small number of patients who are candidates for immunotherapy (7, 8), treatment is largely empiric, with guideline-concordant care driving clinical recommendations and being considered the gold standard (9). While guideline-concordant care can include precision or personalized medicine, a one-size-fits-all approach to empiric guidelines leaves much to be desired in terms of individual patient benefit and patient expectations (10–12).

As physicians and researchers, we need to ask ourselves: Is the standard of care enough?

In this commentary, we describe a hypothetical patient journey for a patient with colon cancer, highlighting common difficulties that hinder the ability to provide personalized medicine.

### 1.1 Patient journey

We describe a clinically grounded hypothetical journey of a patient with colorectal cancer. Our hypothetical patient is a 55-year-old woman who has recently undergone her first screening colonoscopy. She has no family history of colon cancer; however, she has a 3-cm polyp in her right colon that was biopsied and found to be cancerous. She is called 5 business days later and informed that she has invasive colonic adenocarcinoma (1, 13). She is also informed that she has been referred to her local university hospital’s colon cancer multidisciplinary clinic (MDC) (14). Although she is extremely concerned, she feels somewhat comforted by the university’s reputed academic expertise and the promptness of her referral for treatment (15).

She arrives for an early morning appointment, at which time her full medical history is taken and physical examination is performed by a mid-level provider or a junior-level surgery or medicine resident (a doctor in training). Following her history and physical examination, she is quickly taken to the laboratory for a full panel of electrolytes, complete blood count, and “tumor markers.” From the laboratory, she undergoes a CT scan of her chest, abdomen, and pelvis. She efficiently completed the current patient intake requirements (14, 16, 17). At this point, she will return for an afternoon appointment to discuss her case.

Our patient is considering a litany of questions regarding her cancer stage (18), her surgery, and whether she will require chemotherapy or radiation (19). Her questions have been gleaned from Google searches (20) and well-meaning advice from family, friends, and acquaintances who have had experiences with colon cancer or who hold personal opinions on medical procedures (21).

At a planned lunch, a large group of physicians, mid-level providers, residents, interns, medical students, and other assorted ancillary personnel are reviewing and discussing the cases to be seen in the clinic that afternoon. From her intake, it has been discovered that our patient has no evidence of metastatic or regional disease on her axial imaging, no family history, no mitigating medical issues, is eligible for no study protocols, and has no abnormal tumor markers detected in her pathology review to suggest she is a candidate for immunotherapy.

It is therefore recommended that she have upfront surgery via a minimally invasive technique that promises limited pain and a rapid discharge from the hospital (22, 23). Having returned to the clinic, she is greeted by the provider she met that morning, along with a more senior physician who will be her surgeon. The surgeon delivers a brief introduction, explains the plan of care, and asks if our patient has any questions.

The patient begins by asking, “what stage cancer do I have?” (18). This is a common question and, in many ways, demonstrates the lack of pre-clinic preparation patients receive as they go through the initial intake process (15, 24, 25).

In the best of circumstances, her stage question leads to a discussion of the tumor, lymph nodes, and metastatic disease (TNM) staging system and the need for surgery to remove and further evaluate her tumor. The risks and benefits of the surgery, along with a brief mention of the complications, will be followed by the signing of a surgical consent, and she will be placed on the surgery schedule.

At some point in this process, the patient will likely hear that she will be receiving “guideline-concordant care” (14, 16, 17).

It is unclear whether most patients understand what this means (26). This phrase has undoubtedly been mentioned at a physician lunch while reviewing pathology slides and CT scans. Within a few days to weeks of this visit, our patient will return to the hospital to undergo surgical segmental resection of a portion of her colon. Presumably, the surgery went well and was also guideline-concordant (27).

At this point, our patient has her clinical staging completed (28). For the sake of this commentary, we will assume that she has had no unexpected metastases seen during her operative intervention, nor has visible evidence of T4 disease (cancer penetrating the wall of the colon) been detected. Based on her preoperative testing, she has stage 1–3 colon cancer, pending her formal pathology evaluation.

As previously mentioned, she has no family history or other contributory factors that would be considered “high risk” for systemic recurrence. At this point, she is again told that she will be recommended to undergo guideline-concordant care once her pathology is known, which might include chemotherapy.

Our patient is doing well from a surgical standpoint and is told that within 72 h she will be discharged to complete her recovery at home (29).

In this hypothetical scenario, our patient will have her case reviewed at a multidisciplinary tumor board, and a discussion of her next steps in care will take place. Once again, her plan of care should be guideline-concordant.

For the purposes of this hypothetical case, our patient’s pathology has returned as a T2, N0, MX moderately differentiated adenocarcinoma. Based on the existing guidelines (14) for early-stage disease, she will be recommended to undergo surveillance, whereas more advanced disease would elicit a recommendation for adjuvant therapy (30, 31).

In this scenario, the patient will be informed that her chance of survival with surgery alone is quite high (around 90%) (32). Unfortunately, there is a small, but real chance that her cancer can recur, and therefore ongoing surveillance is important.

### 1.2 Recurrence, shortcomings, and potential paths to improvement

The above hypothetical patient presentation leaves us to consider whether or not we have done enough. It has clearly been demonstrated that multidisciplinary clinics are very efficient in seeing patients and getting them into treatment (10), and while this appears to cover all the bases, it is certainly not without issues (9, 33–35).

In terms of providing personalized care, undoubtedly, there is a need for improvements (10, 36). It is known that, even in early-stage patients, a low, but real, risk of metastasis exists (37). The incidence of recurrent disease is higher in stage 3 and 4 colorectal cancer patients and in patients who develop colorectal cancer at a younger age (38–40).

A recent publication from Denmark highlights some challenges in recommending the best next step in treatment for colon cancer patients with non-metastatic disease. This study examined a large number of patients with stage 1–3 disease and noted recurrence rates of more than 16% in stage 1 patients at 1 year, decreasing to approximately 7% at 3 years postoperatively. For stage 2 patients, the rates were approximately 22% at 1 year, dropping to nearly 12% at 3 years, and finally, for stage 3 patients, the rates were 35.5 and 24.6% at 1 and 3 years, respectively (39).

While lower rates are found in other studies (41, 42), there is still a small percentage, but a large corresponding number of early-stage colon cancer patients who develop metastatic disease that might benefit from systemic therapies. Similarly, not all patients with stage 3 disease go on to develop metastases and therefore will not benefit from standard systemic therapies as guidelines recommend (42–44).

When we examine current guidelines, there are a limited number of options for adjuvant therapy (essentially combinations of standard chemotherapeutic agents: capecitabine, 5-fluorouracil (5-FU), and oxaliplatin) (14, 28). The recommendations are anchored primarily in the MOSAIC Trial (45).

In this trial, a total of 1,123 patients were randomized, and the rate of disease-free survival at 3 years was 78.2% (95% confidence interval, 75.6–80.7) in the group given 5-FU/leucovorin plus oxaliplatin and 72.9% (95% confidence interval, 70.2–75.7) in the 5-FU/leucovorin group (*p* = 0.002).

While this is highly significant, and the toxicity can generally be managed (6, 46), recent work suggests that patients may be more enthusiastic about increased survival from adjuvant chemotherapy than clinicians (47). More recently, it has been shown, among more aged patients, that the expectation of prolonged survival needs to be higher for them to accept the risks of adjuvant therapy (48).

The real-world benefits of increased survival balanced against long-term toxicity (peripheral neuropathy, depression, and poor sleep quality) may not be appreciated by patients, particularly as symptoms may commence after treatment is completed (49–51).

Thus, we note that with the use of current guidelines, there is potential to under-treat early-stage colon cancer patients and over-treat a percentage of more advanced stage patients for whom surgery alone would be sufficient. This again leads us to ask, “Is current practice enough?”.

Improvements in selecting patients for receipt of adjuvant therapy, as well as candidacy for upfront small-molecule inhibitors, immunotherapy, or even more efficient surveillance, definitely need to be addressed in a “guideline-concordant” approach, suggesting that we need new data.

### 1.3 Colorectal cancer in the ‘omics era

Genomic profiling has undeniably had a transformative effect in the field of oncology. The proportion of cancer patients receiving genomically guided treatment and the number benefiting from it have been increasing over the past two decades.

One pan-cancer study reported that the percentage of patients benefiting from such treatments increased from 0.7% in 2006 to 4.9% in 2018 (52), while another study published in 2024 reported that 31.6% of tumors carried a biomarker considered predictive in standard-of-care (53).

A recent CRC-specific study of 575 patients reported a rich landscape of potentially clinically actionable genomic findings, including many well-known driver mutations. Ultimately, they suggested that 51% of patients could theoretically be eligible for clinical actionability based on genomic findings, while 49% could be enrolled in clinical trials of investigational therapies.

Nonetheless, this does not represent a real-world benefit, due to the current entrenchment of more traditional treatment paradigms. This fact is not without justification, since the clinical actionability of a mutation does not guarantee successful treatment.

One recent pan-cancer meta-analysis of approximately 55,000 tumors reported that of the 15% of patients who received a genomically guided treatment, the objective response rate was only 25%.

While genomically guided treatment has been, and will continue to be, a driver of improved cancer care, it is only one piece of the required treatment armamentarium, and crucial elements are still missing. One such missing element is the transition of functional precision medicine approaches into standard clinical care (54).

### 1.4 From classical to modern functional precision medicine

Functional precision medicine has existed in some form since the mid-1900s (55) and exposes patient-derived tissues to drugs in an attempt to predict clinical response *ex vivo* (56). It encompasses traditional approaches, including 2D monolayer and clonogenic drug sensitivity assays, as well as more contemporary methods, including patient-derived xenografts (PDXs), patient-derived organoids, and emerging microphysiologic (e.g., microfluidic or tumor-on-chip) systems.

Early methods such as clonogenic assays seemed initially promising but were ultimately abandoned (57) for various reasons, including long assay times, prohibitive biomass requirements, limited testing scope, growth artifacts, and irreproducibility (58). Other highlighted issues included low evaluation rates (57), labor-intensiveness (57), prohibitive assay times, lack of scalability (59, 60), and inability to correlate with clinical response (61).

While PDX models have provided valuable insights into tumor biology and therapeutic response, they are associated with limitations, including months-long engraftment times, the presence of murine components, a lack of a functional human immune system, and clonal evolution that may alter microenvironmental interactions and genomic fidelity relative to the tumor of origin. They are also resource-intensive and relatively low-throughput compared to *ex vivo* systems (62–65).

Microphysiologic systems offer improved biomimicry compared to traditional models; however, these platforms remain largely experimental, technically complex, and relatively low-throughput, which may limit clinical deployment in the short term (66–69). Despite the existence of some commercial tests (55), no methods are widely accepted by the medical community (62).

In recent years, the field has been transitioning toward alternative models that can exhibit the multifactorial biomimicry of an individual’s tumor. Three-dimensional patient-derived tumor organoids (PDTOs) have been demonstrated to maintain features of the original tumor, including intratumoral heterogeneity, architecture, and polyclonality, and are thus regarded as a possible approach to overcoming the shortcomings of traditional methods (66).

Organoids have been shown to retain the histological features, hotspot mutations, and receptor status of the tumor of origin (70), and dose–response studies have demonstrated that organoids can mirror the sensitivity profile of a patient (71, 72). In colorectal cancer specifically, clinical studies have shown wide-ranging success in clinical correlation studies (73).

Organoid assays are not without their own challenges. Differences in sample storage, media composition, cell procurement, and experimental protocols all represent sources of potential experimental variability (74); however, it is believed that industrial developments such as automation and miniaturization will be key in addressing many challenges associated with traditional assays (75–79) and will facilitate clinically compatible turnaround times, which are necessary for widespread adoption of any assay (80).

Ultimately, the success of PDTO-based drug screening in personalized cancer care will require a rapid turnaround from tumor sampling to treatment recommendations to guide clinical decisions (81, 82). The specific promise of organoids in clinical care has been evidenced and strengthened by the National Institutes of Health’s recent announcement of its Standardized Organoid Modeling Center. The goal of the center is the development of standardized organoids and associated protocols for deployment in biological and medicinal research (83).

Across the field, standardized prospective performance metrics, validation frameworks, regulatory pathways, and workflow integration into multidisciplinary oncology practice remain areas of active development. Our own approach, described below, represents one highly automated strategy with the potential to play a role in this evolving landscape. Like other platforms, it will require prospective validation and demonstration of reproducibility and clinical utility before broader adoption can be realized; however, we believe preliminary evidence offers hope for our own or similar technologies to realize clinical benefits.

### 1.5 Clinical correlation and resistant subclone identification in a clinical CRC study

In a recent publication, we demonstrated an association between *ex vivo* testing of patient-derived tumor models and patient clinical response to treatment in a retrospective clinical study incorporating a cohort of 21 colorectal cancer patients treated with neoadjuvant standard-of-care therapies.

While our cohort was modestly sized, and follow-up work, including prospective validation and independent replication of outcomes, is required to confirm and quantify clinical utility, our initial findings were both encouraging and hypothesis-generating.

Patient response to treatment was modeled using PDTOs and our proprietary MOSgen™ system, and the model was tested for correlation with actual clinical outcomes. *Ex vivo* testing was retrospectively associated with clinical response using binary patient response categorization with 83% accuracy for the 21-patient cohort, and disease-free survival was also correlated with in-assay measured responses.

Furthermore, we identified patients whose PDTOs showed higher responses to alternative standard-of-care therapies than to the one they received in their clinical treatment, inspiring the preliminary hypothesis that such patients might have benefited from a non-empiric treatment selection paradigm (84).

The assay used in the aforementioned study utilized brightfield microscopy imaging of approximately 40 individual patient-derived models (MOS® droplets) per well in 384-well plates, with wells spanning multiple drugs and 9-point concentration gradients.

Fluorescently labeled epithelial cell adhesion molecule (EpCAM) was used as a marker of tumor cell viability, with signal intensity measured longitudinally across a 1-week period. While clinical correlation was based on the summation of well-level EpCAM signals, we also utilized machine learning techniques to automatically identify all individual MOS droplets per well and subsequently generated per-MOS-droplet viability measures.

**Figure 1:**
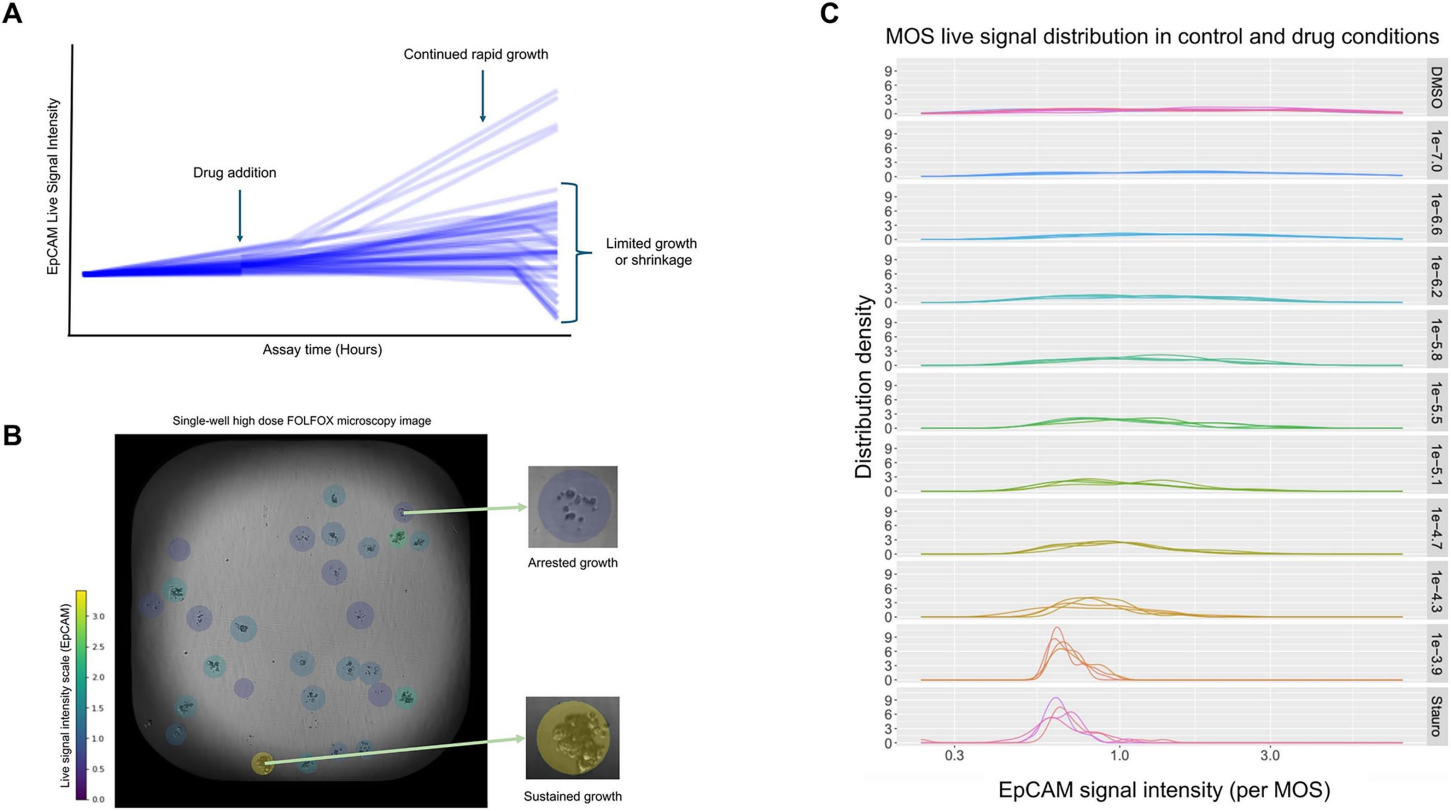
**(A)** Representative example of longitudinal live signal intensity in a drug-treated well for a patient sample. Each line represents a single patient-derived model (MOS droplet). Some natural growth-rate heterogeneity is observed throughout the assay, but post-drug addition, distinct populations of MOS droplets are observed, including those with limited growth, death, and, in select instances, continued rapid cell growth. **(B)** Representative brightfield microscopy image visualizing tumor model heterogeneity. A single high-dose FOLFOX-treated well containing independent patient-derived CRC models (MOS droplets) overlaid with a color gradient indicating high-to-low viability measured by EpCAM fluorescence signal intensity. While the majority of MOS droplets demonstrate low viability, an outlier shows continued, sustained growth suggestive of potential treatment resistance. **(C)** Representative single-patient (MOS model) EpCAM signal intensity distributions. Vehicle (top) and kill control (bottom) wells are shown, with increasing doses of a single treatment (rows 2–10). Wider distributions in vehicle and lower drug doses correspond to higher overall levels of growth heterogeneity. Peaks at higher drug doses correspond to decreased heterogeneity in viability, with the majority of MOS models undergoing growth arrest or death and only outlier MOS models retaining high viability, suggestive of potential treatment resistance.

This provided the ability to detect differential growth and response rates for each individual patient MOS model within each well of a plate, possibly corresponding to the well-documented heterogeneity of cancer cells *in vivo* (Figure 1A).

By combining per-MOS-droplet EpCAM signal measurements with a colored intensity gradient and per-well brightfield microscopy images, we were further able to visualize the morphology of MOS models demonstrating heterogeneous responses to drug doses, with some outlier MOS models demonstrating vigorous, sustained growth despite high-dose drug treatment, while others appeared arrested in growth and condensed in terms of cellular morphology (Figure 1B).

EpCAM signal density distributions per-well were also profiled and demonstrated wider distributions corresponding to higher overall levels of growth heterogeneity in vehicle or lower drug-dose containing wells, while heterogeneity decreased with high drug doses, and high viability was observed only in isolated outlier MOS models.

We hypothesize that such outliers might represent drug-resistant subclonal populations within the patient tumor; however, this interpretation remains hypothetical since follow-up orthogonal validation was not part of the study (Figure 1C).

While this analysis was exploratory and the clinical study was retrospective, it suggests that, with further work, there is potential for technologies like ours to accurately predict patient treatment sensitivities in a prospective setting and, further, to identify clinically relevant, heterogeneous intra-patient drug responses.

The latter would require follow-up validation work to generate evidence supporting the clinical relevance of differential drug responses *ex vivo*. This could include comparative ‘omics profiling of outlier MOS models to their drug-responsive counterparts for identification of known or novel drivers of resistance, or isolation of non-responsive MOS models followed by secondary or combination treatment response profiling.

Such efforts could offer the prospect of identifying sub-clonal drug resistance and even potentially determining alternative, accompanying, or second-line agents that might evoke cell death in initially resistant subpopulations of cells from a single patient assay early in a patient’s clinical journey.

Further, identification of such subclones could introduce the possibility of more efficacious treatments in all stages of colorectal cancer. Such isolation of identified subclones would offer a focused alternative to traditionally used pan-tumor assessment (85, 86), enabling genetic analysis of the most problematic cancer-cell populations. While the potential seems high, whether this strategy ultimately translates into improved clinical outcomes will require prospective evaluation in appropriately designed studies.

## 2 Conclusion

There have been advances in the care of colorectal cancer patients, including enhanced recovery after surgery and minimally invasive surgery, with the promise of reduced pain and earlier hospital discharge (87, 88).

There have been advances in our ability to measure minimal residual disease in colon cancer (89, 90). More recently, there have been exciting advances in immunotherapy for appropriately selected patients (83).

Unfortunately, guideline-concordant therapy for non-metastatic colon cancer revolves around systemic therapy recommendations based on data that are two decades old (87).

At the same time, the aged, frail population continues to increase (91), and more young patients are presenting with aggressive colon cancers than ever before (92).

It is our belief that current paradigms of colon cancer treatment may benefit from the near-term incorporation of personalized medicine approaches.

The work of Nors et al. (40) has demonstrated that recurrence rates for non-metastatic colorectal cancer, particularly at 1–2 years, are much higher than previously quoted.

We believe such findings may help explain unexpected disease recurrence in early-stage disease as well as treatment failure in patients receiving standard-of-care systemic therapy for more advanced disease.

Our preliminary work in MOS models, created from primary colon cancers, suggests that problematic subclones that do not respond to standard-of-care systemic therapy may be present and identifiable in *ex vivo* models (Figures 1B,C).

It is our belief that the identification of treatment-resistant cell niches may offer the potential for fastidious use of ‘omics by focusing investigation on problematic subclones rather than addressing potentially billions of cells in a primary tumor.

Further, we feel this study could enable improved diagnostics for a wide population of cancer patients.

Ultimately, the role of functional precision medicine in colorectal cancer will need to be determined by prospective validation and thoughtful integration alongside existing standards of care.

It is our opinion that continued investigation will help define its contribution to more individualized clinical care.

## Data Availability

The original contributions presented in the study are included in the article/supplementary material, further inquiries can be directed to the corresponding author.
